# MBL-1 and EEL-1 affect the splicing and protein levels of MEC-3 to control dendrite complexity

**DOI:** 10.1371/journal.pgen.1010941

**Published:** 2023-09-20

**Authors:** Jianxin Xie, Wei Zou, Madina Tugizova, Kang Shen, Xiangming Wang

**Affiliations:** 1 National Laboratory of Biomacromolecules, CAS Center for Excellence in Biomacromolecules, Institute of Biophysics, Chinese Academy of Sciences, Beijing, China; 2 College of Life Sciences, University of Chinese Academy of Sciences, Beijing, China; 3 The Fourth Affiliated Hospital, Zhejiang University School of Medicine, Yiwu, China; 4 Institute of Translational Medicine, Zhejiang University, Hangzhou, China; 5 Howard Hughes Medical Institute, Department of Biology, Stanford University, Stanford, California, United States of America; 6 Department of Cell Biology, School of Basic Medical Science, Capital Medical University, Beijing, China; University of California San Diego, UNITED STATES

## Abstract

Transcription factors (TFs) play critical roles in specifying many aspects of neuronal cell fate including dendritic morphology. How TFs are accurately regulated during neuronal morphogenesis is not fully understood. Here, we show that LIM homeodomain protein MEC-3, the key TF for *C*. *elegans* PVD dendrite morphogenesis, is regulated by both alternative splicing and an E3 ubiquitin ligase. The *mec-3* gene generates several transcripts by alternative splicing. We find that *mbl-1*, the orthologue of the muscular dystrophy disease gene muscleblind-like (MBNL), is required for PVD dendrite arbor formation. Our data suggest *mbl-1* regulates the alternative splicing of *mec-3* to produce its long isoform. Deleting the long isoform of *mec-3(deExon2)* causes reduction of dendrite complexity. Through a genetic modifier screen, we find that mutation in the E3 ubiquitin ligase EEL-1 suppresses *mbl-1* phenotype. *eel-1* mutants also suppress *mec-3(deExon2)* mutant but not the *mec-3* null phenotype. Loss of EEL-1 alone leads to excessive dendrite branches. Together, these results indicate that MEC-3 is fine-tuned by alternative splicing and the ubiquitin system to produce the optimal level of dendrite branches.

## Author summary

Dendrite morphology is essential for the proper functioning of neurons. Abnormalities in dendrite structure are associated with different neurological disorders like autism, schizophrenia, and Rett’s syndrome. Using forward genetic approaches, we identify two new factors, alternative splicing regulator MBL-1 and E3 ubiquitin ligase EEL-1, which participate in regulating PVD dendrite morphogenesis in *C*. *elegans*. MBL-1 regulates the alternative splicing of transcription factor MEC-3 to produce its long isoform. EEL-1 reduces the amount of MEC-3 protein to ensure its correct expression level. Our findings indicate that the combination of alternative splicing and ubiquitin ligase on the level of a transcription factor to regulate dendrite morphogenesis, which is unexpected and original. Both *mbl-1* and *eel-1* are genes associated with diseases, and the transcription factor MEC-3 may act as their target, which will provide insight into the mechanism of these diseases.

## Introduction

Transcription factors (TFs) control gene expression and are essential for specifying different aspects of neuronal cell fate, including neurotransmitter phenotypes [1], neurite morphogenesis and membrane excitability [2–4]. Many TFs have been reported to affect dendrite morphogenesis [5–7]. For example, dendrite branching of vertebrate cortical neurons requires the notch signaling and NFkappaB [8]. Activity regulated transcription factor CREB is critical for neuronal activity induced dendritic growth and branching [9]. The homeobox TF Cux1 is required for dendritic arborization in specific cortical neuron populations [10]. In *Drosophila*, *hamlet* represses complex dendritic arbor and promotes single-dendrite morphology [11]. The development of the dendrites in the Da classes of neurons also requires other families of TFs including Abrupt, Knot, and spineless [12–14]. Different expression levels of the TF Cut specify the complexity of dendritic arborization in the four different classes of Da Neurons.

The PVD sensory neurons in *C*. *elegans* elaborate highly stereotyped and branched dendrites [15]. The PVD cell body sends one anterior and one posterior primary dendrite (1°). The secondary dendrites (2°) grow orthogonally from 1° dendrites in both ventral and dorsal directions. Upon reaching the body wall muscle borders, 2° branches turn anterior and posterior then form tertiary branches (3°) along the sub lateral nerve cords. From the 3° dendrites, numerous quaternary dendrites (4°) grow orthogonally away from the lateral midline [16]. PVD neurons sense harsh touch and cold temperature and act as nociceptors [16]. They also function as proprioceptive sensors to regulate body bend amplitude [17]. Developmentally, the complex and stereotyped dendrites are guided by the neuronal receptor DMA-1 and the epidermal adhesion molecule complex SAX-7-MNR-1-LECT-2 [18–23]. The elaborate dendritic arbor of the PVD also requires the LIM homeobox TF MEC-3 and the POU domain transcription factor UNC-86 [15,24,25]. In addition, AHR-1/spineless suppresses the dendrite branching program and prevents other neurons to adopt the PVD like dendritic arbors [25].

Alternative splicing regulates activities of transcription factors by inclusion or exclusion of exons. For example, Srp is a transcription factor involved in *Drosophila* mesoderm development. Two isoforms, SrpC (exclusion of N-finger) and SrpNC (inclusion of N-finger) are generated by alternative splicing, which differentially stimulate the expression of *crq* and *gcm*, respectively [26]. Muscleblind-like proteins (MBNL) belong to a family of RNA splicing regulators of precursor mRNA [27]. They regulate alternative splicing by promoting inclusion or exclusion of specific exons on different pre-mRNAs through antagonizing the activity of CUG-BP and ETR-3-like factors (CELF proteins) [28]. Pathological CUG and CCUG expansion sequesters MBNLs, leading to loss of MBNLs activity and causes myotonic dystrophy (DM) [29]. While MBNLs clearly regulate many muscle genes, in recent years, MBNLs/MBL-1 are reported to be associated with neuronal development [30–32]. However, the underlying mechanisms are not understood.

Huwe1 is a highly conserved member of the HECT E3 ubiquitin ligase family and functions in many cellular processes such as cell proliferation/suppression, embryogenesis and apoptosis [33]. In recent years, Huwe1 has been shown to degrade the N-Myc oncoprotein during nervous system development [34]. EEL-1 (the orthologue of Huwe1 in *C*. *elegans*) regulates GABAergic presynaptic transmission in *C*. *elegans* [35]. Human genetic studies show that Huwe1 is associated with multiple neurodevelopment disorders, such as X-linked mental retardation (XLMR) [36]. However, little is known about the downstream targets of Huwe1/EEL-1 in neuronal development and disorders.

Here we report that alternative splicing and proteasome degradation system contributes to the precise regulation of the key TF MEC-3. MBL-1 regulates the alternative splicing of *mec-3* to produce the MEC-3 isoform with a higher branching promoting activity. Additionally, EEL-1 may directly or indirectly downregulate MEC-3’s protein level to maintain its proper expression level for PVD dendrite morphogenesis. These results demonstrate that the precise control of MEC-3 at mRNA and protein level are required for proper dendrite morphogenesis.

## Results

### *mbl-1* mutants disrupt PVD dendrite morphology

**Fig 1:**
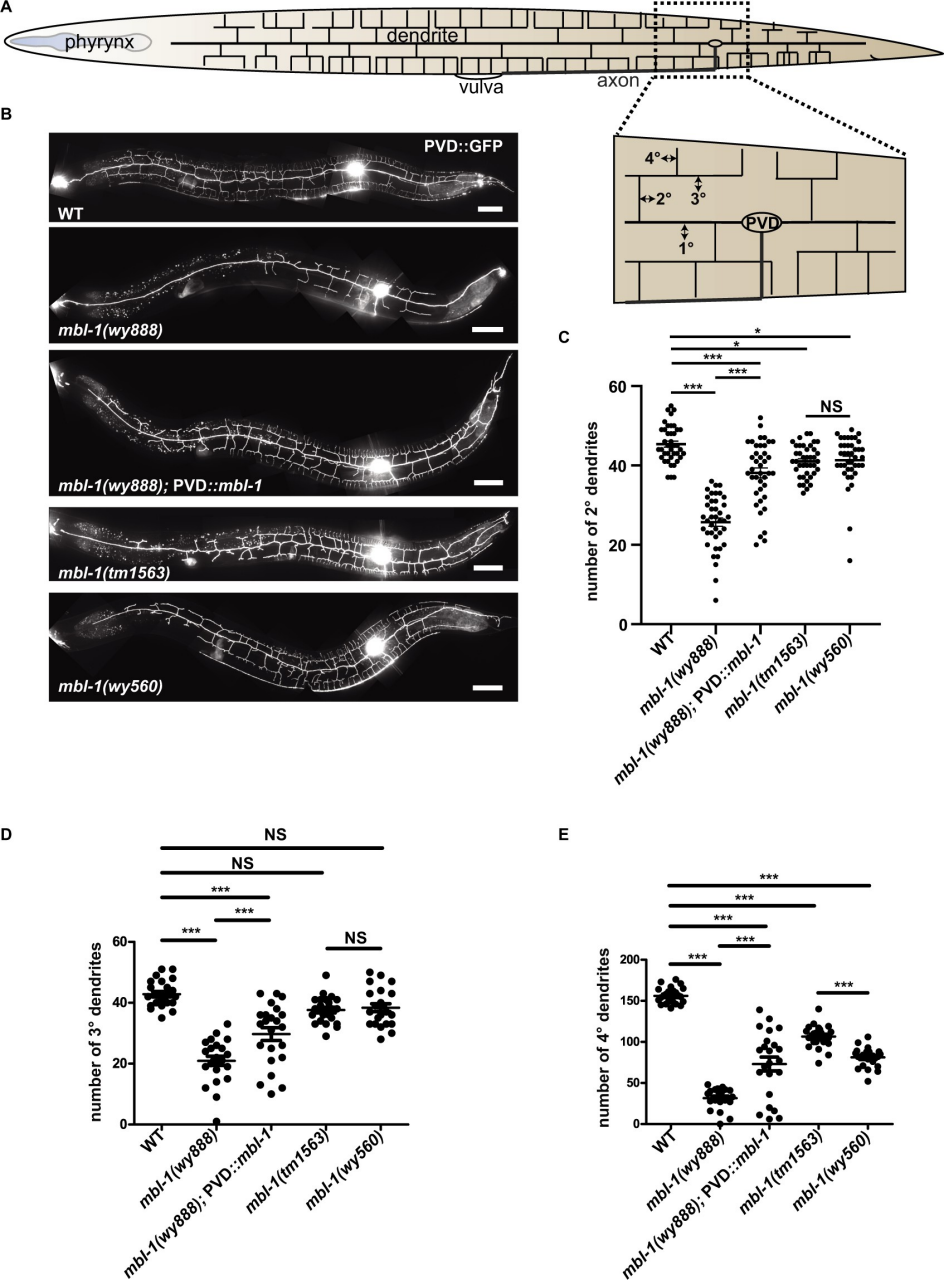
*mbl-1* is required for PVD dendritic morphogenesis. (A) A cartoon showing the morphology of the PVD dendritic arbor. Primary dendrites (1°), secondary dendrites (2°), tertiary dendrites (3°), quaternary dendrites (4°). (B) Representative confocal images showing the PVD dendrite pattern, illustrated by *ser-2Prom3*::GFP, of young adult wild type (WT), *mbl-1(wy888)*, *mbl-1(wy888);* PVD::*mbl-1*, *mbl-1(tm1563)*, and *mbl-1(wy560)* animals. Scale bars, 50 μm. (C-E) Quantification of the number of 2°, 3°, and 4° dendrites in WT, *mbl-1(wy888)*, *mbl-1(wy888)*; PVD::*mbl-1*, *mbl-1(tm1563)*, and *mbl-1(wy560)* animals. Data are shown as mean ± SEM. One-way ANOVA with Tukey’s multiple comparisons test. Not significant (NS) p>0.05, *p<0.05, ***p<0.001. n>20 for each genotype.

To understand molecular mechanisms regulating PVD dendrite morphology, we carried out a candidate screen using GFP labeled PVD neuron (Fig 1A and 1B). We identified a background mutation *wy888* in the strain RB1712, which showed drastically reduced dendrites and simplified arbors (Fig 1B). Through genetic mapping and sequencing, we identified the causative gene for *wy888* to be *mbl-1*. A single point mutation was found in *mbl-1*, which leads to amino acid substitution of the conserved amino acid histidine to tyrosine (H131Y) (S1 Fig). Expression of wild type *mbl-1* cDNA under a PVD specific promoter could rescue *wy888* phenotype, indicating *mbl-1* mutant causes the dendrite reduction phenotype and *mbl-1* functions cell autonomously in PVD (Fig 1B–1E). To further examine this idea, we analyzed two other putative null alleles of *mbl-1*, *tm1563* (a 513-bp deletion that eliminates the exon 3 of *mbl-1*) and *wy560* (a 70-kb deletion that eliminates eight genes, one of which is *mbl-1*). The data showed that both of them led to PVD dendrite reduction phenotype (Fig 1B–1E). Interestingly, dendrite phenotype of *wy888* is more severe than the two putative null alleles. The mutation in the *wy888* allele (H131Y) is a conserved residue located in the zinc-knuckle-like motif CCCH (C3H) that is required for mRNA binding [37]. This point mutation might affect binding to its target mRNA and prevent the access of its target mRNA to other splicing factors.

### MBL-1 regulates the alternative splicing of *mec-3*

To identify the downstream factors of MBL-1, we constructed double mutants of *mbl-1* with genes involved in PVD dendrite morphogenesis including *hpo-30*, *dma-1*, *tiam-1*, *ire-1*, and *kpc-1* (S2A Fig). Unlike the other double mutants, *ire-1; mbl-1* double mutant did not show a more severe dendrite phenotype compared to that of the *mbl-1* single mutant, suggesting that *ire-1* may be a splicing target of MBL-1. Therefore, we examined the cDNAs of *ire-1* in WT and *mbl-1(wy888)* mutants and found no differences between these two genotypes (S2B and S2C Fig). Therefore, we do not have evidence that *ire-1* is a splicing target of *mbl-1*.

**Fig 2:**
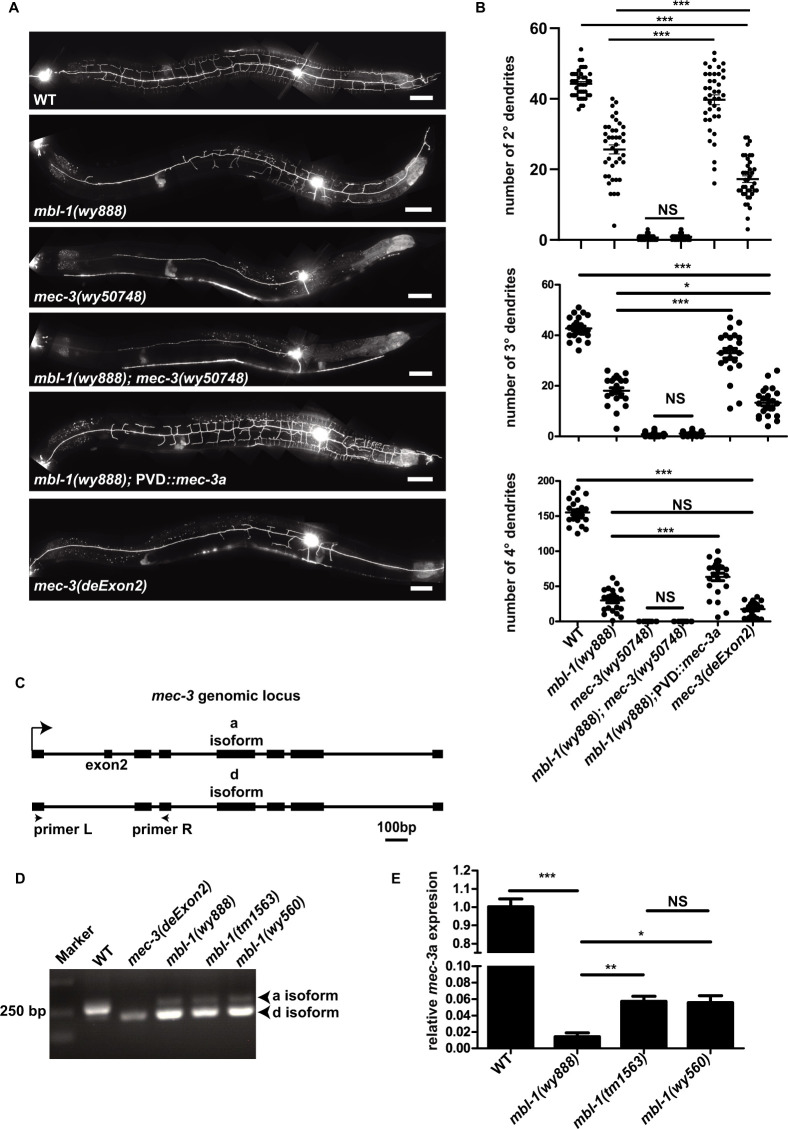
*mbl-1* controls PVD dendrite morphology through regulating the *mec-3* exon2 exclusion. (A) Representative confocal images showing the PVD dendrite pattern in WT, *mbl-1(wy888)*, *mec-3(wy50748)*, *mbl-1(wy888); mec-3(wy50748)*, *mbl-1(wy888);* PVD::*mec-3*a (cDNA), *mec-3(deExon2)*. Scale bars, 50 μm. (B) Quantification of 2°, 3°, and 4° dendrite number in WT, *mbl-1(wy888)*, *mec-3(wy50748)*, *mbl-1(wy888); mec-3(wy50748)*, *mbl-1(wy888);* PVD::*mec-3*a (cDNA), and *mec-3(deExon2)* animals. Data are shown as mean ± SEM. One-way ANOVA with Tukey’s multiple comparisons test. Not significant (NS) p>0.05, ***p<0.001. n>20 for each genotype. (C) A schematic diagram showing the *mec-3* genomic locus. *mec-3*a isoform: exon2 inclusion. *mec-3*d isoform: exon2 exclusion. For *mec-3*a isoform, using primer (L+R), RT-PCR products is 267 bp. While for the exon2 lacking d isoform, the RT-PCR products is 216 bp. (D) A representative gel of the RT-PCR products amplified from the cDNA of WT, *mec-3(deExon2)*, *mbl-1(wy888)*, *mbl-1(tm1563)*, and *mbl-1(wy560)* animals. (E) qPCR results of *mec-3*a in WT, *mbl-1(wy888)*, *mbl-1(tm1563)*, and *mbl-1(wy560)* animals.

MEC-3 functions as the vital TF to control essential downstream targets for PVD dendrite development. In *mec-3* null mutant, the highly branched PVD dendritic arbors are nearly completely lost with only the unbranched primary dendrite (Fig 2A and 2B). This phenotype was similar, albeit stronger, as the phenotype of the *mbl-1* mutants (*wy888*, *tm1563*, *wy560*), suggesting they might function in the same pathway. To test this idea, we constructed the double mutants between *mec-3* and *mbl-1* and the data showed that *mbl-1* could not enhance *mec-3* phenotype (Fig 2A and 2B). This result is consistent with the notion that they function in the same genetic pathway, and raises the hypothesis that *mec-3* might be the splicing target of *mbl-1*.

To test if there are changes of the *mec-3* transcripts in the *mbl-1* mutant, we designed primers to examine the alternative splicing of *mec-3*. Comparing to the wild type, the *mbl-1* mutant showed different band sizes for fragments near the 5’ end of the *mec-3* transcript. Specifically, in wild type the longer transcript, corresponding to the *mec-3*a isoform, was brighter, while the shorter transcript, corresponding to the *mec-3*d isoform, was dimmer. In *mbl-1(wy888)* mutant, the shorter transcript becomes the dominant species (Fig 2D). Sequencing experiments showed that the difference between the two isoforms was that the second exon was included in the longer *mec-3*a isoform and spliced out for the shorter *mec-3*d isoform. The second exon is 51 base pair long and would not create frameshift when it is missing. The corresponding protein sequence locates at the N-terminus of the protein, in the first LIM domain (S3 Fig), which is known to be a protein-protein interaction domain. Consistently, the two null alleles of *mbl-1* mutant showed similar RT-PCR gel pattern (Fig 2D). To quantitatively assess the splicing defects, we used qRT-PCR to measure the level of the *mec-3*a isoform in WT, *mbl-1(wy888)*, *mbl-1(tm1563)*, and *mbl-1(wy560)* animals. The level of *mec-3*a isoform in *wy888* allele is less than 2% of the wild type control, while the two null alleles have about 6*%* of the level found in the control (Fig 2E). This is consistent with the finding that the *wy888* allele of *mbl-1* showed more severe PVD dendrite phenotype than the two null alleles. These data suggest that *mbl-1* promotes the *mec-3*a isoform and inhibits the *mec-3*d isoform. To test if MBL-1 affects known MEC-3’s transcriptional targets, we used qRT-PCR to measure the levels of *acp-2*, *hpo-30*, *T24F1*.*4*, and *egl-46* [25]. The level of *egl-46* transcripts is significantly lower in *mbl-1(wy888)* mutant (S4D Fig), whereas the other three (*acp-2*, *hpo-30*, *T24F1*.*4*) did not show significant decrease (S4A–S4C Fig). The reason might be that *acp-2*, *hpo-30*, *T24F1*.*4* expressed in other cells beyond MEC-3-expressing cells (a few neurons) and controlled by additional transcription factors. We constructed *mbl-1(wy888); egl-46(gk692)* double mutants, the results showed that it could not enhance the phenotype of *mbl-1(wy888)* (S5A Fig), which further suggests that *egl-46* and *mbl-1* are in the same pathway. Overexpressing *egl-46* cDNA in PVD neurons of *mbl-1(wy888)* mutants could rescue the phenotype slightly (S5B Fig), which support that EGL-46 functions downstream of MBL-1.

Because *mbl-1* likely regulates many mRNAs, we wonder whether the reduced *mec-3*a is largely responsible for the PVD dendrite phenotype in the *mbl-1* mutants. We used two approaches to test this idea. First, we expressed the *mec-3*a cDNA in the *mbl-1(wy888)* mutant background using a PVD specific promoter. Remarkably, this transgene largely rescued the *mbl-1* mutant dendrite phenotype (Fig 2A and 2B), suggesting the *mbl-1(wy888)* mutant phenotype is mainly caused by the dramatically lowered level of *mec-3*a. It is reported that *mbl-1*/MBNL1 functions through consensus binding sequence GCUU [38]. In addition, we found 1 GCUU in the first intron and 3 GCUU in the second intron of *mec-3*, suggesting the cis-elements might be conserved.

**Fig 3:**
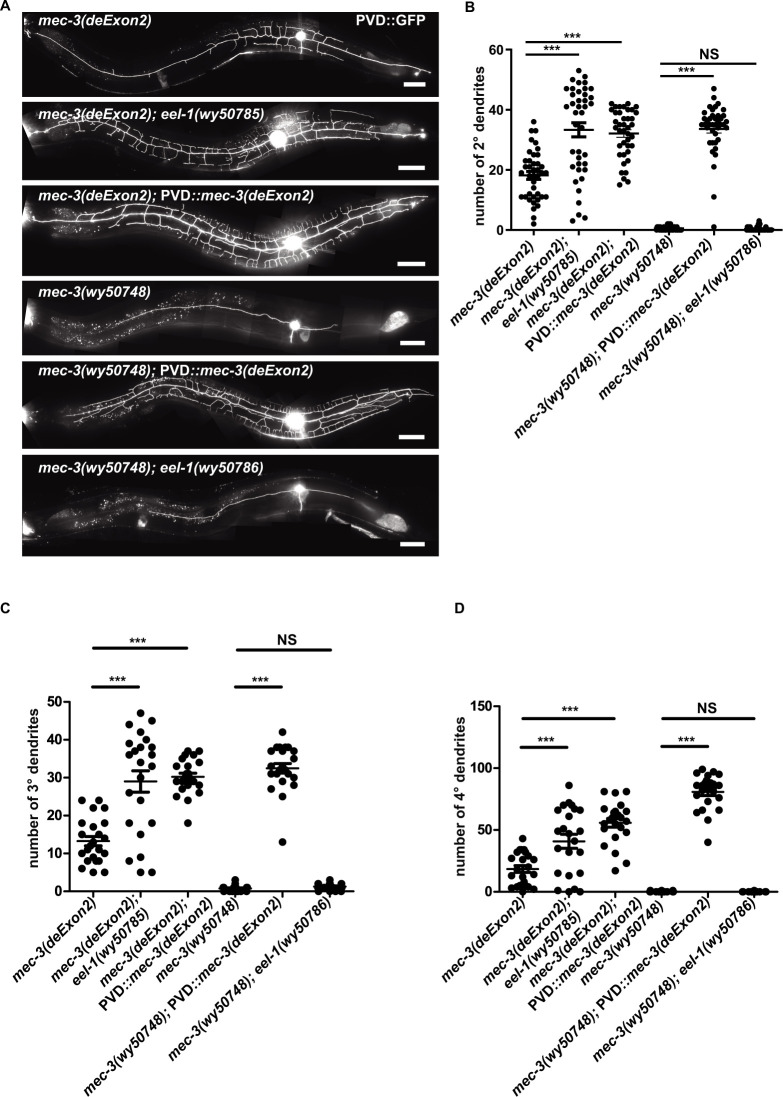
EEL-1 maintains the normal PVD dendrite through MEC-3. (A) Representative confocal images showing the PVD dendrite pattern in *mec-3(deExon2)*, *mec-3(deExon2); eel-1(wy50785)*, *mec-3(deExon2);* PVD::*mec-3(deExon2)* cDNA, *mec-3(wy50748)*, *mec-3(wy50748);* PVD::*mec-3(deExon2)*, *mec-3(wy50748); eel-1(wy50786)*. Scale bars, 50 μm. (B-D) Quantification of 2°, 3°, and 4° dendrite number in *mec-3(deExon2)*, *mec-3(deExon2); eel-1(wy50785)*, *mec-3(deExon2);* PVD::*mec-3(deExon2)*, *mec-3(wy50748)*, *mec-3(wy50748)*; PVD::*mec-3(deExon2)* cDNA, and *mec-3(wy50748); eel-1(wy50786)*. Data are shown as mean ± SEM. One-way ANOVA with Tukey’s multiple comparisons test. Not significant (NS) p>0.05, ***p<0.001. n>20 for each genotype.

Second, we used CRISPR-Cas9 to generate a *mec-3(deExon2)* allele by deleting the second exon of *mec-3*. By RT-PCR assay, we verified that this allele completely lacked *mec-3*a (Fig 2D). Indeed, *mec-3(deExon2)* showed dramatic reduction of dendrite complexity. This phenotype was stronger than the *mbl-1* mutants but was not severe as the *mec-3* null mutants (Fig 2A and 2B). These results are consistent with the fact that there are still 2–6% of the *mec-3*a transcripts left in the *mbl-1* mutants. Together, these data indicate that the MEC-3a isoform carries the majority of MEC-3’s activity and that the lack of the *mec-3*a isoform in *mbl-1* mutants account for their dendrite phenotypes. Compared with *mec-3* null mutant, *mec-3(deExon2)* PVD could still elaborate some dendrite branches, suggesting that MEC-3d is partially active, likely with lower activity than MEC-3a. Indeed, overexpression of PVD driven MEC-3(deExon2) rescued the *mec-3(deExon2)* and CRISPR constructed *mec-3(wy50748)* null phenotype, suggesting that MEC-3d carries partial transcription factor activity, albeit lower than MEC-3a (Fig 3A–3D).

In contrast to the highly branched dendrites of PVD, the other MEC-3 expressing neurons have simple, non-branched dendrites and therefore are not suitable for studying complex dendrite morphogenesis. To test if *mbl-1* functions in other MEC-3 expressed neurons, we crossed *mbl-1(wy888)* into *zdIs5* strain, which labelled the touch receptor neurons (AVM and PVM). The results showed that *mbl-1* mutant did not affect the neurite morphology of AVM and PVM neurons (S6 Fig). It suggests that AVM and PVM might only requires a very low functional MEC-3 or *mbl-1* might not function in these neurons at all.

### E3 ligase EEL-1 mutant suppresses *mbl-1(wy888)* mutant phenotype not through splicing *mec-3*

**Fig 4:**
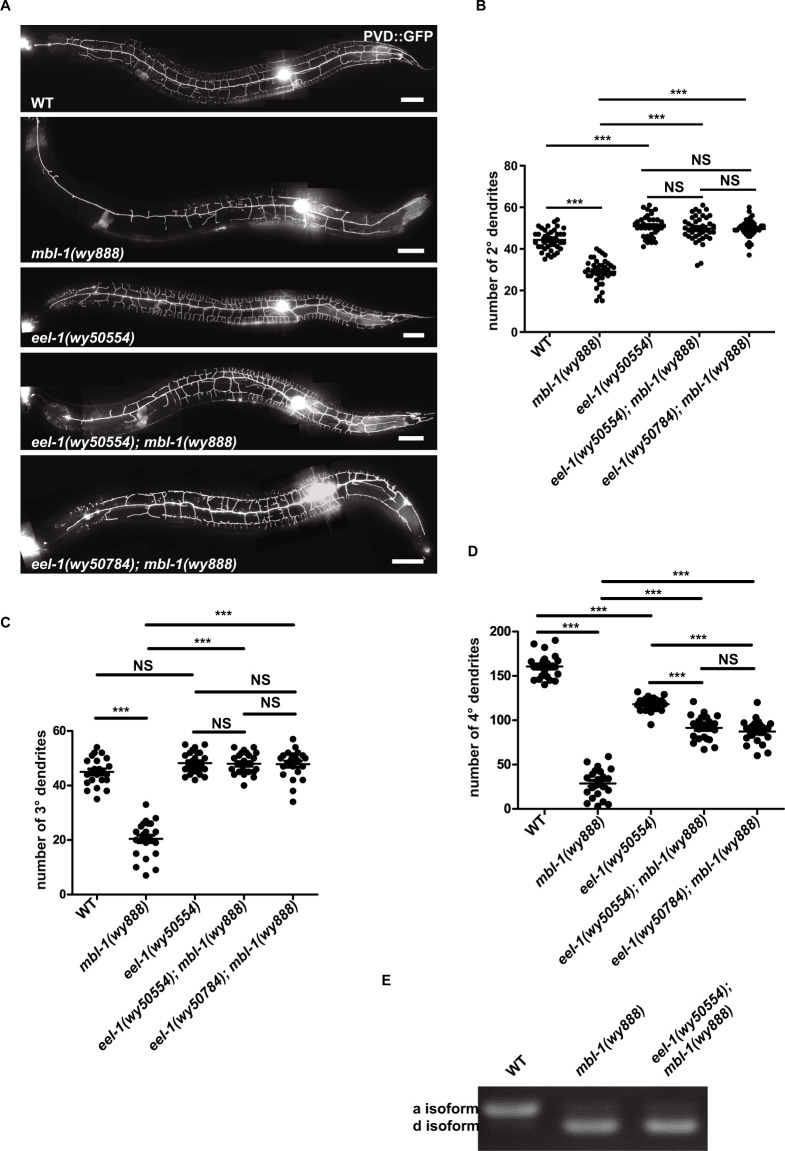
E3 ligase EEL-1 mutant suppresses *mbl-1(wy888)* phenotype. (A) Representative confocal images showing the PVD dendrite pattern in WT, *mbl-1(wy888)*, *eel-1(wy50554)*, *eel-1(wy50554); mbl-1(wy888)*, *eel-1(wy50784); mbl-1(wy888)*. Scale bars, 50 μm. (B-D) Quantification of 2°, 3°, and 4° dendrite number in WT, *mbl-1(wy888)*, *eel-1(wy50554)*, *eel-1(wy50554); mbl-1(wy888)*, and *eel-1(wy50784); mbl-1(wy888)*. Data are shown as mean ± SEM. One-way ANOVA with Tukey’s multiple comparisons test. Not significant (NS) p>0.05, ***p<0.001. n>20 for each genotype. (E) A representative gel of the RT-PCR products amplified from the cDNA of WT and *eel-1(wy50554); mbl-1(wy888)* animals.

To further understand the genetic program that regulates the *mbl-1/mec-3* pathway, we performed a modifier screen on the *mbl-1(wy888)* mutant. From a 3000 haploid genome screen, we isolated one allele *wy50554*, which suppressed the dendrite reduction phenotype of *mbl-1(wy888)* (Fig 4A–4D). Through genetic mapping and high-seq assay, we identified a G-to-A point mutation, which causes a glutamic acid to lysine substitution in the conserved acidic domain (CAD) of the *eel-1* gene. The point mutation changed an acid amino acid to alkaline amino acid (E2475K), which might disrupt the CAD function. To confirm the *eel-1(wy50554)* mutation is responsible for the dendrite phenotype, we constructed a putative null allele *wy50784* (S7 Fig) of *eel-1* using CRISPR-Cas9. Indeed, *eel-1(wy50784); mbl-1(wy888)* exhibited the same rescued dendrite branches as *eel-1(wy50554); mbl-1(wy888)* (Fig 4A–4D), indicating that inactivating *eel-1* suppresses the *mbl-1(wy888)* phenotype.

As *mbl-1* regulates *mec-3* pre-mRNA splicing, the possible mechanism of *eel-1* is to regulate *mec-3* pre-mRNA splicing process. To examine this idea, we performed RT-PCR using *eel-1(wy50554); mbl-1(wy888)* cDNA. Interestingly, similar to *mbl-1(wy888)*, the *eel-1(wy50554); mbl-1(wy888)* double mutants showed dramatically reduced *mec-3*a and increased *mec-3*d transcripts (Fig 4E), indicating *eel-1* inhibition did not correct the splicing deficit in the *mbl-1* mutants.

We overexpressed *mec-3*a isoform (full-length) cDNA in the PVD neurons of WT worms. The overexpression resulted in subtle but statistically significant increase in the number of 2°, 3°, and 4°dendrites (S8A Fig). *mec-3*a overexpression in *eel-1(wy50554)* mutation background did not further increase the dendrite branching (S8B Fig), suggesting that there is a ceiling effect of the level of MEC-3.

### EEL-1 might degrade MEC-3 protein to maintain the normal PVD dendrite

To test if EEL-1 functions through MEC-3, we constructed *mec-3(deExon2); eel-1(wy50785)* (S7 Fig) double mutants and found that *eel-1(wy50785)* could suppress *mec-3(deExon2)* phenotype (Fig 3A–3D), suggesting *eel-1* functions through *mec-3*. This result implies that EEL-1 may directly or indirectly regulate MEC-3 protein level. Hence, in the *eel-1* mutant, it is plausible that the level of MEC-3d in the *mec-3(deExon2)* mutant is increased, leading to the rescue of dendrites, although this notion needed to be further confirmed.

EEL-1, a conserved E3 ubiquitin ligase, and its mammalian homolog Huwe1 have been shown to degrade TFs such as P53 and SKN-1 [39,40]. Therefore, it is conceivable that EEL-1 may degrade MEC-3 to control PVD dendrites. This hypothesis could explain the suppression of the *mbl-1(wy888)* phenotype by *eel-1* mutations. As in *mbl-1(wy888)* mutant, the exon2-lacking *mec-3*d isoform is the main form, and protein level increase of the partially functional MEC-3(deExon2), caused by *eel-1* mutant, rescued *mbl-1(wy888)* mutant phenotype. This is the most plausible explanation for the suppression effect of *eel-1* mutant, however we still cannot completely rule out other possibilities. Actually, overexpression of PVD driven MEC-3(deExon2)/*mec-3*d cDNA rescued the *mec-3(deExon2)* and *mec-3(wy50748)* null phenotype (Fig 3A–3D), confirming that the protein level of MEC-3(deExon2) is positive correlation with the dendrite complexity. If EEL-1 degrades MEC-3 to control PVD dendrite morphology, we predict that *eel-1* mutant could not suppress the *mec-3(wy50748)* null phenotype as no MEC-3 or partial functional MEC-3(deExon2) exists. Indeed, *mec-3(wy50748); eel-1(wy50786)* double mutant exhibited the same phenotype as *mec-3(wy50748)* alone (Fig 3A–3D), supporting the idea that EEL-1 degrades MEC-3. To further support this hypothesis, we examined the expression level of known MEC-3 transcription targets. As expected, all the four genes (*acp-2*, *hpo-30*, *T24F1*.*4* and *egl-46*) expression increased in *mbl-1(wy888)*; *eel-1(wy50554)* double mutants (S4A–S4D Fig).

To further test if EEL-1 functions as the E3 ligase to degrade MEC-3, we used CRISPR to disrupt the function of the HECT domain, which is the catalytic ubiquitin ligase domain. We isolated one frameshift mutation, a single base deletion (A11171 deletion), in *mec-3(deExon2)* mutant background. This mutation is immediately upstream of HECT domain and should cause a complete deletion of the HECT domain (S7 Fig). The dendrite phenotype of *mec-3(deExon2)* mutant was significantly suppressed (S9 Fig), indicating that the HECT ubiquitin ligase domain may be essential for *eel-1*’s function in suppressing *mec-3(deExon2)* phenotype, although we are not sure if this truncation mutant might affect EEL-1 protein stability or expression levels. The possibility of non-ubiquitin ligases mechanisms for EEL-1 function in shaping dendrite complexity can still not be ruled out.

### *eel-1* mutants increase the endogenous MEC-3 protein level in PVD neurons

**Fig 5:**
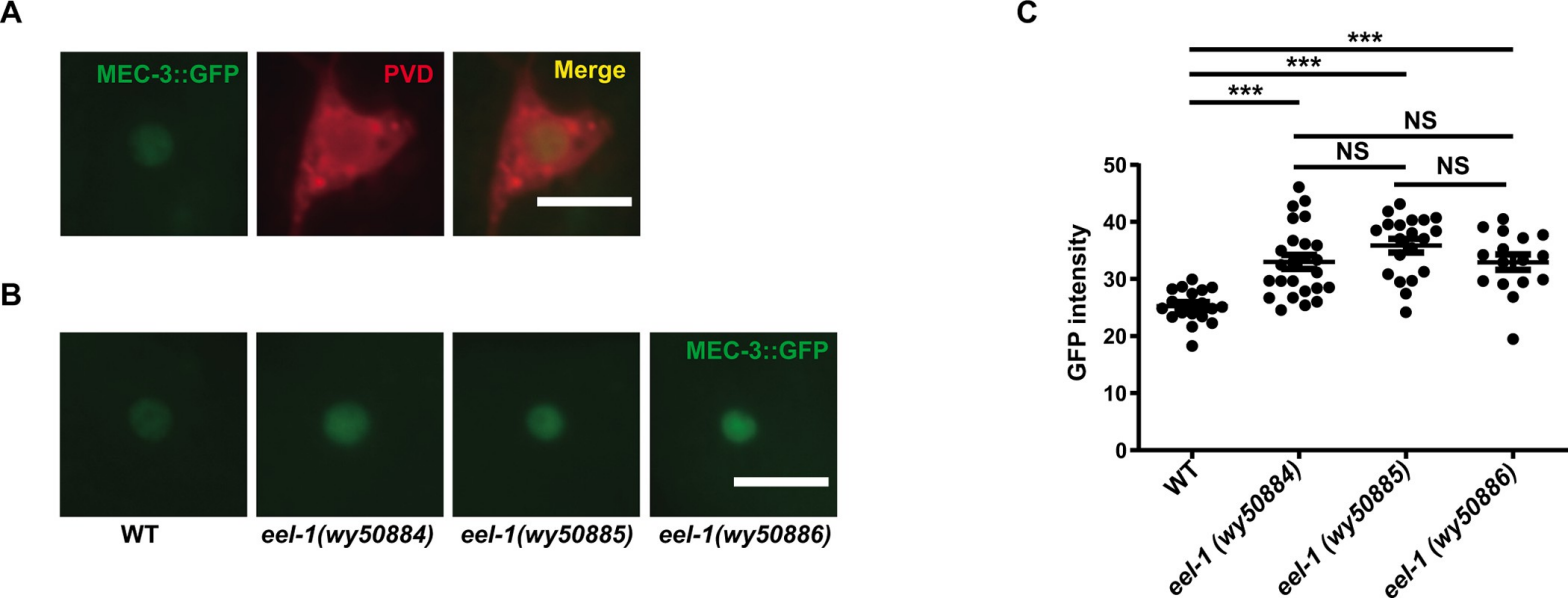
The E3 ligase EEL-1 regultes MEC-3 level in PVD neuron. (A) Subcellular localization of MEC-3::GFP and PVD marker (*ser-2Prom3*::mCherry) in wild-type animals. Scale bar, 10 μm. (B) MEC-3::GFP protein levels of PVD neurons in WT and three independent *eel-1* knock out animals. Scale bar, 10 μm. (C) Quantification of GFP fluorescence intensity in WT and the three independent *eel-1* knock out animals. Data are shown as mean ± SEM. One-way ANOVA with Tukey’s multiple comparisons test. Not significant (NS) p>0.05, ***p<0.001. n>15 for each strain.

To further verify the idea that EEL-1 controls PVD dendrite morphology through degrading MEC-3 proteins, we generated an endogenous MEC-3::GFP (GFP fused to MEC-3 before stop codon) knockin strain using the CRISPR-Cas9 technique. As expected, MEC-3::GFP localized in PVD nucleus (Fig 5A). To test if *eel-1* mutants modulate endogenous MEC-3 protein level, we created three independent *eel-1* knock out alleles (*wy50884*, *wy50885*, *wy50886*) using CRISPR-Cas9 (S7 Fig) in this MEC-3::GFP knockin strain background. Comparing the GFP fluorescence intensity with wild type, we found that the MEC-3::GFP signal was increased in all three independent *eel-1* knock out strains (Fig 5B and 5C). This result is consistent with our hypothesis that the putative ubiquitin ligase EEL-1 downregulates MEC-3 to control PVD dendrite morphogenesis.

## Discussion

While it is well appreciated that TFs specify dendrite morphology, the regulation of the TFs themselves is not well understood. In *C*. *elegans*, the key TF MEC-3 directs several downstream proteins such as HPO-30 and EGL-46 to shape PVD dendrite. How MEC-3 is regulated at mRNA and protein level is not clear.

We identified a background mutation named *mbl-1(wy888)* from RB1712 strain. Further investigation reveals that MBL-1 regulates the alternative splicing of *mec-3*. First, *mbl-1* could not enhance *mec-3* mutant phenotype but it does enhance *hpo-30*, *dma-1*, *tiam-1*, *and kpc-1* phenotype (S2A Fig), suggesting *mbl-1* functions in the same pathway as *mec-3*. Interestingly, we also found that *mbl-1* could not enhance *ire-1* mutant phenotype, however, we did not observe *ire-1* splicing defect in *mbl-1* mutant (S2B and S2C Fig). Second, the full length *mec-3* cDNA (a isoform) rescued *mbl-1* phenotype largely, suggesting *mec-3* is the main downstream target of *mbl-1* although we could not preclude any other putative targets. Third, RT-PCR results demonstrated that the ratio between the *mec-3* a isoform and d isoform (lacking the second exon) was reversed with higher level of d isoform and nearly null a isoform in *mbl-1* mutant, suggesting *mbl-1* regulates the alternative splicing of the *mec-3* second exon. Fourth, the orthologue of MBL-1 functions as alternative splicing factor and *mbl-1* is reported to splice *sad-1* in worm neuron [38]. Although it has been well reported that MBLN1 directly interacts with the pre-mRNA of its splicing targets in other models [41], there is still no evidence that MBL-1 binds *mec-3* pre-mRNA directly in *C*. *elegans*. Interestingly, MBL-1 is also a downstream target of MEC-3 [38], indicating that a positive feedback exists between the two genes to pattern PVD dendrite.

Interestingly, the *wy888* allele shows stronger phenotype than the two putative null alleles. The *wy888* point mutation locates at H131, which is the conserved C3H motif binding zinc ion. One possible explanation is that the H131Y mutation likely impairs zinc binding and then affects binding between *mec-3* mRNA and MBL-1(H131Y). In the two *mbl-1* null alleles, MBL-1 protein is completely absent while MBL-1(H131Y) protein might still bind to *mec-3* mRNA and block access of other alternative splicing factors. In fact, a previous paper showed that the C3H-type zinc finger modules containing protein Makorin competes with Bru1 for binding to the substrate *osk* mRNA [42].

Why is *mbl-1* mutant phenotype weaker than *mec-3* null mutant? We propose that the second exon lacking isoform *mec-3*d exhibits partial function of the full length *mec-3*a isoform. First, the exon 2 specific knock-out allele *mec-3(deExon2)* was weaker than *mec-3* null allele, but stronger than *mbl-1(wy888)*, which is probably caused by the few *mec-3*a isoform in *mbl-1(wy888)*. Second, overexpression of *mec-3(deExon2)* construction could rescue *mec-3(deExon2)* and *mec-3* null allele to a large extent, indicating that although the second exon-lacking *mec-3*d isoform is not potent as the *mec-3*a isoform, it does show partial function and can compensate the *mec-3*a isoform function when overexpressed.

Besides regulation at the mRNA level, our results also show that EEL-1, the E3 ligase, downregulates the activity of MEC-3d possibly through proteostasis, but further biochemical studies are needed to determine if MEC-3 is a definitive EEL-1 substrate. First, *eel-1* suppressed *mbl-1* mutant phenotype, suggesting it functions in the same pathway as *mbl-1*. Then it could also suppress *mec-3(deExon2)* phenotype, suggesting it functions through *mec-3*. Second, *eel-1* could not suppress *mec-3* null phenotype. The possible explanation is that in *mec-3(null)*; *eel-1* double mutants, no MEC-3 protein is produced and the *eel-1* mutation could not increase MEC-3 level when there is no MEC-3. Therefore, *eel-1* mutation could not suppress the phenotype of *mec-3(null)*, although we could not completely exclude other possibilities. Third, *eel-1* mutant exhibited more branches than the wild type, reminiscent of high level of MEC-3 protein, suggesting EEL-1 degrades MEC-3 to shape PVD dendrite. Further biochemical experiments are still needed to strengthen this hypothesis.

**Fig 6:**
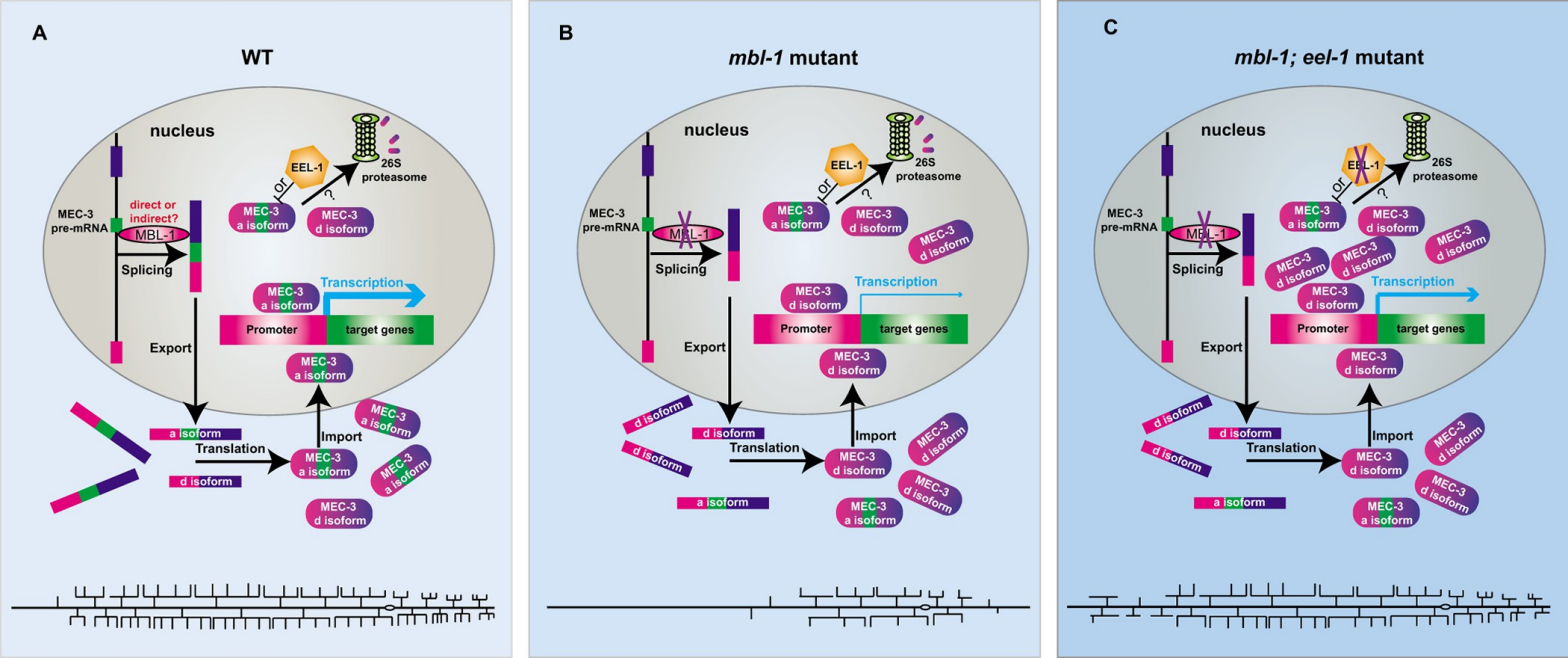
Schematic model of the regulation mechanisms of dendrite morphogenesis in *C*. *elegans* PVD neuron. (A) In wild-type animals, MBL-1 regulates the alternative splicing of *mec-3*, producing the great majority of *mec-3*a isoform and very few of the *mec-3*d isoform mRNAs, therefore promotes the normal dendrite matures. (B) In *mbl-1* mutant, the ratio of *mec-3*a isoform and d isoform changes in that the d isoform is predominant, hampering the transcriptional efficiency of *mec-3* downstream targets, ultimately decreasing dendrite complexity significantly. (C) In *mbl-1* and *eel-1* double mutants, the increased MEC-3d isoform quantity due to the E3 ligase EEL-1 mutant, although with lower transcriptional activity, restores the dendritic arbors to a WT-like morphology. Alternatively, EEL-1 functions indirectly to regulate MEC-3.

During PVD development, the essential TF MEC-3 should be tightly controlled. The splicing factor MBL-1 splices the second exon of *mec-3* to provide enough potent *mec-3*a isoform to produce full functional TF MEC-3. At the same time, the MEC-3 level should be balanced by the E3 ligase EEL-1 to keep normal MEC-3 protein level, avoiding excessive branching of PVD. These elegant regulation mechanisms at post-transcriptional and post-translational ensure the proper level of MEC-3 to pattern appropriate dendrites of PVD (Fig 6A–6C).

Many neuronal diseases have been reported to be associated with MBL-1 and EEL-1, and our findings, which the TF MEC-3 might function as their target, will enlighten the mechanism of these diseases.

## Materials and methods

### *C*. *elegans* strains and genetics

*C*. *elegans* strains were cultured at 20°C using nematode growth medium (NGM) plates. *Escherichia coli* OP50 was seeded in NGM plates. The reference strain is wild-type N2. All the mutants and transgenic strains were genetically modified based on N2.

### Isolation and mapping of mutants

We isolated *wy888* from RB1712 strain. Based on *mbl-1(wy888); wyIs592*, we isolated *wy50554* by a modifier screen of 3000 haploid genomes. 50mM ethyl methanesulfonate (EMS) was used as mutagen in these two screen processes. Genetic mapping and sequencing showed that the *wy888* mutant is a C-to-T point mutation in exon 4 of the *mbl-1* gene, which changes a histidine to a tyrosine (c.C391T:p.H131Y). In addition, the *wy50554* mutant is a G-to-A point mutation in the exon 8 of the *eel-1* gene, which changes a glutamic acid to a lysine (c.G7423A:p.E2475K). SNP mapping was performed using standard methods [43].

### Constructs and transgenes

Plasmid constructs were generated in pPD95.77 vector. The Clontech In-Fusion PCR Cloning System was used for vector construction and these constructs were verified by sequencing to ensure the correct results. The PCR products were amplified with Phusion DNA polymerase (New England Biolabs) or TransStart FastPfu DNA Polymerase (Transgen Biotech) by standard procedures. Standard microinjection techniques were used to generate the transgenic worms. The DNA plasmids were injected into N2, *mbl-1(wy888)*, *wyIs592*, *mec-3(deExon2)*, and *mec-3(wy50748)* hermaphrodites at a concentration from 10 to 50 ng/ml. 2 ng/ml P*myo-2*::mCherry plasmids were used as the co-injection marker. Knockout and knockin worms were generated by standard CRISPR/Cas9 technique. We generated *mec-3(deExon2)* allele by co-injection of the mixture of P*eft-3*_*mec-3*_gRNA (CRISPR-Cas9 plasmids) and repair templates, deleting the entire exon2 of *mec-3* in the genome.

### Fluorescent imaging

Young adult animals were anesthetized with 1 mg/ml levamisole in M9 buffer before mounted on 3% (w/v) agar pads. Images of the *mec-3*::GFP and the three corresponding independent *eel-1* KO worms were captured in live animals at the same exposure time (2000 ms) using a 63× objective by AxioImager M2 microscope (Carl Zeiss). And images of PVD dendritic arbors of the related animals were captured by the spinning-disk confocal imaging system which includes an Axio Observer Z1 microscope (Carl Zeiss MicroImaging) equipped with a 40× objective, an electron-multiplying charge-coupled device camera (Andor), and the 488- and 568- nm lines of a Sapphire CW CDRH USB Laser System attached to a spinning-disk confocal scan head (Yokogawa CSU-X1 Spinning Disk Unit). Micro-Manager (https://micro-manager.org) software and ImageJ (http://rsbweb.nih.gov/ij/) software were used to process the images.

### qRT-PCR

RNA was isolated by TriPure Isolation Reagent (Roche, catalog no.93996120) from the whole worm of mixed stages and reverse-transcribed using RT reagent kit (TaKaRa, catalog no. RR047A). mRNA expression was measured by qRT-PCR using the ΔΔCT method with the following primers: *mec-3*a: (forward primer, ATGGAAATGTTAGAGTCAAAG; reverse primer, CATAAATTTGCTCATTGCAGC), *act-1*: (forward primer, CCAGGA

ATTGCTGATCGTATG; reverse primer, GGAGAGGGAAGCGAGGATAG). *acp-2*: (forward primer, GAGTATCCAGAAGGGAGAAG; reverse primer,

TTAGCTCATGATCCTCGGCAG), *hpo-30*: (forward primer, AGATGAAGAGGCCAGAGAGC; reverse primer, GAACATGCTCCGGTCATAAAG), *T24F1*.*4*: (forward primer, CATTCGGCTAAGCAGACAAG; reverse primer, CAAAACGGCGGCGAGTAATAG), *egl-46*: (forward primer, TGTTCTGGAACCCAACGCTAG; reverse primer, GACTGGAGAACTGGTCACAG). Data are presented as mean ± SEM, and Student’s t-test (two-tailed distribution, two-sample unequal variance) was used to calculate P-values. Statistical significance is displayed as Not significant (NS) p>0.05, *p<0.05, **p<0.01, ***p<0.001. The tests were performed using Graphpad Prism.

### Quantification and statistics

The number of 2°, 3°, and 4° dendrites of young adults was counted under the microscope using a 40× objective. The MEC-3::GFP mean intensity in the nucleus was measured by ImageJ software. All statistical tests were performed using one-way ANOVA with Tukey’s multiple comparisons test in Graphpad Prism.

## Supporting information

S1 Fig Sequence alignment of zinc finger domains between the *C*. *elegans* MBL-1, *Drosophila* muscleblind, and human muscleblind-like protein families. The two C3H-type zinc finger domains are marked by underlines. A single point mutation in *mbl-1(wy888)* leads to histidine to tyrosine (H131Y) substitution.
(EPS)

S2 Fig Genetic analysis of *mbl-1* and genes regulating PVD dendrite. (A) Double mutant analysis between *mbl-1(wy888)* and mutants that showed PVD dendrite phenotype. Images showing the PVD dendrite pattern in *mbl-1(wy888)*, *dma-1(wy686)*, *mbl-1(wy888); dma-1(wy686)*, *hpo-30(ok2047)*, *mbl-1(wy888); hpo-30(ok2047)*, *kpc-1(gk8)*, *mbl-1(wy888); kpc-1(gk8)*, *tiam-1(tm1556)*, *mbl-1(wy888); tiam-1(tm1556)*, *ire-1(ok799)*, *mbl-1(wy888); ire-1(ok799)*. Scale bars, 50 μm. (B) A schematic diagram showing the *ire-1* genomic locus. The size of RT-PCR products: primer (L1+R1), 1234 bp; primer (L2+R2), 795 bp; primer (L3+R3), 1009 bp. (C) A representative gel of the *ire-1* RT-PCR products amplified from the cDNA of WT and *mbl-1(wy888)* animals.
(EPS)

S3 Fig The second exon of MEC-3 locates at the highly conserved N-terminal portion of the LIM1 domain. The second exon of *mec-3* corresponds to AA27-43 of the MEC-3a protein. It locates at the N-terminal portion of the LIM1 domain, which is highly conserved. The exon 2 skipping will cause an in-frame deletion, which disrupts the first LIM domain.
(EPS)

S4 Fig Expression level changes of the downstream target genes of MEC-3 in *mbl-1(wy888)* and *mbl-1(wy888); eel-1(wy50554)*. (A) qPCR results of *acp-2* in WT, *mbl-1(wy888)*, and *mbl-1(wy888); eel-1(wy50554)*. (B) qPCR results of *hpo-30* in WT, *mbl-1(wy888)*, and *mbl-1(wy888); eel-1(wy50554)*. (C) qPCR results of *T24F1*.*4* in WT, *mbl-1(wy888)*, and *mbl-1(wy888); eel-1(wy50554)*. (D) qPCR results of *egl-46* in WT, *mbl-1(wy888)*, and *mbl-1(wy888); eel-1(wy50554)*. Data are shown as mean ± SEM. One-way ANOVA with Tukey’s multiple comparisons test. Not significant (NS) p>0.05, *p<0.05, **p<0.01, ***p<0.001.
(EPS)

S5 Fig *egl-46* might function in the downstream of *mbl-1*. (A) Images showing the PVD dendrite pattern in *mbl-1(wy888)*, *egl-46(gk692)*, and *mbl-1(wy888); egl-46(gk692)*. Scale bars, 50 μm. (B) Quantification of the number of 2°, 3°, and 4° dendrites in *mbl-1(wy888)* and *mbl-1(wy888)*; PVD::*egl-46* animals. Data are shown as mean ± SEM. *p<0.05 by unpaired *t* test. n>20 for each genotype.
(EPS)

S6 Fig *mbl-1(wy888)* mutant do not affect the neurite morphology of AVM and PVM neurons. (A) Schematic diagram of AVM and PVM neurons in *C*. *elegans*. (B) Images showing the neurite morphology of AVM and PVM neurons in WT and *mbl-1(wy888)*, respectively. Scale bars, 50 μm.
(EPS)

S7 Fig Diagram of the mutation sites in different *eel-1* alleles. Mutation sites of *eel-1(wy50554)*, *eel-1(wy50784)*, *eel-1(wy50785)*, *eel-1(wy50786)*, *eel-1(wy50884)*, *eel-1(wy50885)*, *eel-1(wy50886)*, and *eel-1(wy50891)* are shown in the diagram.
(EPS)

S8 Fig Overexpressing *mec-3*a cDNA in PVD neurons increases dendrite number of WT, but not *eel-1(wy50554)* mutant, slightly. (A) Quantification of the number of 2°, 3°, and 4° dendrites in WT and PVD::*mec-3*a animals. (B) Quantification of the number of 2°, 3°, and 4° dendrites in *eel-1(wy50554)* and *eel-1(wy50554)*; PVD::*mec-3*a animals. Data are shown as mean ± SEM. Not significant (NS) p>0.05, *p<0.05, **p<0.01, ***p<0.001 by unpaired *t* test. n>20 for each genotype.
(EPS)

S9 Fig The HECT ubiquitin ligase domain might be essential for *eel-1*’s function in suppressing *mec-3(deExon2)* phenotype. (A) Schematic of the *C*. *elegans* EEL-1 protein sequences. Conserved protein domains are annotated as follows: DUF, domain of unknown function; UBA, ubiquitin-associated domain; CAD, conserved acidic domain; HECT, homologous to E6AP c-terminus domain. The HECT domain is the catalytic ubiquitin ligase domain of EEL-1 protein. In *eel-1(wy50891)* mutants, the HECT domain is destructed by a frameshift mutation. (B) Representative confocal images showing the PVD dendrite pattern in *mec-3(deExon2)* and *mec-3(deExon2); eel-1(wy50891)*. Scale bars, 50 μm. (C) Quantification of 2°, 3°, and 4° dendrite number in *mec-3(deExon2)* and *mec-3(deExon2); eel-1(wy50891)*. Data are shown as mean ± SEM. ***p < 0.001 by unpaired *t* test. n>30 for each genotype.
(EPS)
